# Predictors of Postoperative Complications in Metabolic and Bariatric Surgery: A Retrospective Analysis Using Multivariable Logistic Regression

**DOI:** 10.3390/medicina62050881

**Published:** 2026-05-04

**Authors:** Gon Shoham, Shira Naveh, Tariq Zoabi, Noa Gosher, Nir Messer, Jonathan B. Yuval, Mati Shnell, Adam Abu-Abeid

**Affiliations:** 1 Department of Plastic and Reconstructive Surgery, Tel Aviv Sourasky Medical Center, Weizman St. 6, Tel Aviv-Yafo 6423906, Israel; 2 The Gray Faculty of Medical & Health Sciences, Tel Aviv University, Klatzkin St 35, Tel Aviv-Yafo 6997801, Israeljyuval@gmail.com (J.B.Y.);; 3 Division of General Surgery, Tel Aviv Sourasky Medical Center, Weizman St. 6, Tel Aviv-Yafo 6423906, Israel; 4 Gastroenterology Unit, Tel Aviv Sourasky Medical Center, Weizman St. 6, Tel Aviv-Yafo 6423906, Israel

**Keywords:** bariatric surgery, postoperative complications, logistic regression, descriptive analysis, risk stratification

## Abstract

*Background and Objectives*: Metabolic and bariatric surgery is the most effective long-term intervention for severe obesity, associated with significant reductions in weight, associated medical problems, and cancer risk. While the overall safety profile of metabolic and bariatric surgery has improved, early postoperative complications still occur and may lead to prolonged hospitalization, reintervention, or increased morbidity. This study aimed to identify independent preoperative and perioperative predictors of early postoperative complications following metabolic and bariatric surgery. *Materials and Methods*: We conducted a retrospective cohort study of 927 patients who underwent metabolic and bariatric surgery at a single tertiary medical center between December 2017 and March 2022. Early postoperative complications, defined as those occurring during the index hospitalization or within 90 days, were recorded and graded using the Clavien-Dindo classification. Univariate analyses were performed to identify candidate predictors, followed by multivariable logistic regression using an unpenalized model. Odds ratios (ORs) and corresponding 95% confidence intervals (CIs) were estimated using maximum likelihood methods with Wald-based intervals. *Results*: Eighty-four patients (9.1%) experienced postoperative complications, with 38% requiring invasive intervention. Bleeding was the most common complication (46%), followed by leak/intra-abdominal abscess (24%) and cardiorespiratory events (18%). Independent predictors of complications included obstructive sleep apnea (OR: 1.93), bariatric surgery within the past 5 years (OR: 2.39). *Conclusions*: OSA and recent previous surgery increase the risk of early complications after metabolic and bariatric surgery. These findings support integrating specific risk factors into preoperative planning to improve surgical outcomes.

## 1. Introduction

Severe obesity is widely recognized as a global epidemic, with prevalence steadily increasing across nearly all age groups and geographic regions. It is estimated that by the year 2050, nearly two out of every three adults worldwide will be affected [1]. This ongoing rise in prevalence reflects a complex interplay of environmental, behavioral, and biological factors that collectively contribute to excessive weight gain.

This represents a major global health crisis with profound medical, social, and economic consequences. Severe obesity is associated with increased morbidity and mortality due to its well-established causal links to a wide range of chronic diseases. These include type 2 diabetes mellitus, cardiovascular diseases (including ischemic heart disease and stroke), chronic kidney disease, several obesity-related malignancies, osteoarthritis, respiratory insufficiency, and psychological disorders such as depression [2,3,4,5]. The cumulative burden of these conditions places substantial strain on healthcare systems and significantly affects quality of life and long-term health outcomes for affected individuals.

The most effective treatment of severe obesity is metabolic and bariatric surgery [6]. Numerous clinical studies and long-term observational cohorts have demonstrated favorable outcomes following these procedures, including sustained and clinically meaningful weight loss, remission or improvement of obesity-related diseases, and a reduction in obesity-associated cancer incidence [7,8,9,10]. The overall popularity and utilization of metabolic and bariatric surgery have steadily increased in recent years, largely due to its proven effectiveness and its relatively favorable safety profile when compared with other surgical interventions [11]. Bariatric surgery has also been shown to result in significantly higher rates of remission and prevention of type 2 diabetes mellitus, hypertension, and dyslipidemia, while simultaneously improving cardiovascular risk factors and overall quality of life when compared with medical therapy alone [3,9,12].

Perioperative and long-term complications associated with bariatric procedures have decreased substantially since the beginning of the 21st century [13] Ongoing technological improvements, advances in surgical techniques, and refinements in perioperative care have all contributed to a significant reduction in complication rates over the past two decades [14,15,16]. In particular, the transition from open to minimally invasive laparoscopic techniques has significantly reduced perioperative morbidity and mortality while enabling faster recovery and shorter hospital stays [16,17]. The demonstrated safety of these procedures has been a key factor in prompting updated clinical guidelines [18].

Preoperative multidisciplinary evaluation, including nutritional and mental health screening, and rigorous postoperative follow-up for micronutrient supplementation, have further reduced adverse events and improved long-term safety [19]. Enhanced perioperative care pathways and standardized postoperative monitoring have also contributed to improved outcomes in bariatric patients.

Collectively, these advances have led to perioperative mortality rates of 0.1–0.5% and major complication rates of 2–6%, comparable to other elective abdominal procedures [14]. Nevertheless, major complications still occur and can impose a substantial burden on patients. Identifying specific risk factors may aid in reducing their incidence or guiding the implementation of targeted strategies to manage them effectively.

Several studies have investigated predictors of postoperative complications following bariatric surgery, identifying factors such as older age, prior bariatric procedures, and type 2 diabetes [19,20]. However, the relative contribution and clinical significance of these factors remain incompletely understood, and the full spectrum of perioperative predictors has yet to be clearly defined.

The objective of the present study was to identify predictors of early postoperative complications following metabolic and bariatric surgery. Improved risk stratification may enable more effective preoperative optimization, better informed procedure selection, and the development of more individualized perioperative management strategies aimed at improving patient safety and surgical outcomes.

## 2. Materials and Methods

### 2.1. Patients

This retrospective cohort study included all consecutive patients who underwent metabolic and bariatric surgery at a single tertiary university medical center between December 2017 and March 2022. The aim of the study was to evaluate clinical and perioperative factors associated with early postoperative complications following bariatric surgery. Early postoperative complications were defined as adverse events occurring during the index hospitalization or within 90 days following surgery. Clinical data were extracted from the institutional electronic medical records and operative databases. All patients were followed throughout their index hospitalization, during routine postoperative outpatient follow-up visits and emergency referrals. Baseline demographic and clinical variables included age, sex, smoking status, body mass index (BMI), history of previous abdominal or bariatric surgery, and obesity associated medical problems. Comorbid conditions evaluated in this study included hypertension, dyslipidemia, obstructive sleep apnea, gastroesophageal reflux disease, asthma, stress urinary incontinence, and type 2 diabetes mellitus. Perioperative variables included type of bariatric procedure, operative duration, and intraoperative events. Postoperative outcomes included occurrence of complications, need for invasive intervention, and length of hospital stay.

The Clavien-Dindo score was used for complication grading [21]. Non-eligible records, in which more than 10% of the data was missing, were excluded.

### 2.2. Statistical Analysis

Descriptive statistics were calculated to summarize the baseline characteristics of the study population. Continuous variables are presented as mean ± standard deviation, while categorical variables are presented as frequencies and percentages. Variables were classified as binary (e.g., gender, smoking status, and associated medical problems), continuous (e.g., age, body mass index, and surgery length), and categorical (e.g., type of bariatric surgery).

Data preprocessing and analysis were performed using Python (version 3.7) with the SciPy libraries. Missing values in continuous variables were imputed using the column mean. Categorical variables were imputed with “Missing” and then one-hot encoded. Missing data was less than 2% of the entire dataset. Descriptive statistics were generated for each variable, and group comparisons between patients with and without complications were performed using *t*-tests for continuous variables and chi-square tests for categorical variables. *p* values were calculated to assess statistical significance; A *p*-value lower than 0.05 was considered statistically significant.

To identify independent predictors of postoperative complications, we performed multivariable logistic regression using the statsmodels Logit function in Python. An unpenalized logistic regression model was fitted using maximum likelihood estimation. Variables included in the model were selected based on clinical relevance and univariate analysis (*p* < 0.1). Regression coefficients (β) were estimated using maximum likelihood, and standard errors were obtained from the model covariance matrix. Odds ratios (ORs) were calculated by exponentiating the coefficients. Corresponding 95% confidence intervals (CIs) were computed using the Wald method (β ± 1.96 × SE). Model assumptions were assessed by evaluating multicollinearity and overall model fit. A two-sided *p*-value < 0.05 was considered statistically significant.

## 3. Results

### 3.1. Cohort

Between December 2017 and March 2022, a total of 927 patients underwent metabolic and bariatric surgery at our center. Postoperative complications occurred in 84 patients (9.1%) during the index hospitalization.

### 3.2. Univariate Analysis of Complications

Univariate analysis (Table 1) showed that several factors were significantly associated with postoperative complications. These included previous bariatric surgery (25% vs. 36%, *p* = 0.038), prior surgery within the past 5 years (7% vs. 17%, *p* = 0.002), obstructive sleep apnea (OSA) (18% vs. 29%, *p* = 0.023), and borderline significance for pharmacologically treated type 2 diabetes mellitus (17% vs. 26%, *p* = 0.05). Patients with complications were also older (45.3 ± 12.2 vs. 41.4 ± 13.3 years, *p* = 0.01), and more likely to have undergone Roux-en-Y gastric bypass (25% vs. 14%, *p* = 0.011). Surgery duration was also longer in this group (117.4 ± 72.9 vs. 104.9 ± 48.8 min, *p* = 0.034).

### 3.3. Multivariable Analysis

**Figure 1:**
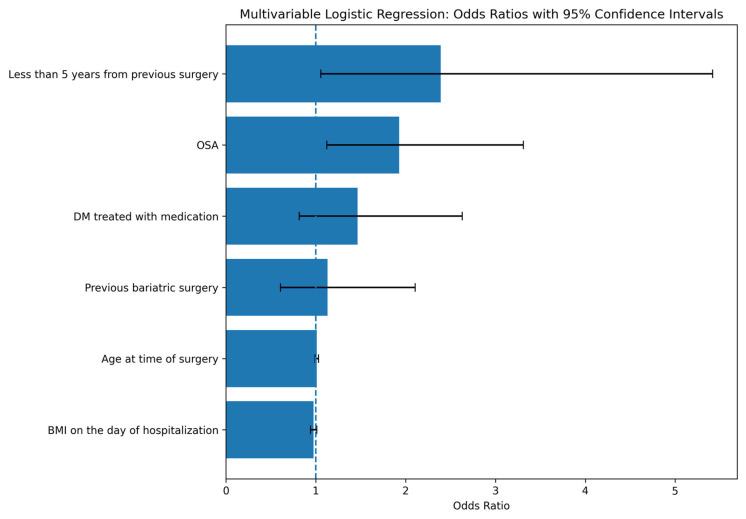
Odds Ratios With 95% Confidence Intervals for Predictors of Postoperative Complications Following Bariatric Surgery. The blue area represents the odds ratio (OR), and the black line represents the 95% confidence interval (CI).

Multivariable logistic regression identified OSA as the strongest independent predictor of postoperative complications, with an odds ratio (OR) of 1.93 (95% CI: 1.1–3.3, *p*-value: <0.05). An additional significant predictor was bariatric surgery within the past 5 years (OR: 2.39, 95% CI: 1–5.4, *p*-value: <0.05). non statistically significant predictors included pharmacologically treated diabetes mellitus (OR: 1.4, 95% CI: 0.81–2.6, *p*-value: 0.2), age (OR: 1.00, 95% CI: 0.99–1.03, *p*-value: 0.25), BMI (OR: 0.97, 95% CI: 0.94–1.01, *p*-value: 0.17), and previous bariatric surgery (OR 1.1, 95% CI 0.6–2.1, *p*-value: 0.69). The estimated odds ratios and corresponding 95% confidence intervals are presented in Figure 1.

The distribution of complications, classified by Clavien-Dindo grade [15], was as follows: grade I in 51 patients (60% of complications; 5.5% of the total cohort), grade II in 9 patients (11%; 0.97%), grade III in 15 patients (18%; 1.94%), and grade IV in 9 patients (11%; 0.97%). The most common complication was postoperative bleeding, affecting 39 patients (46% of those with complications; 4.2% of the total cohort). Leak or intra-abdominal abscess was observed in 20 patients (24%), of which 5 (6%) were classified as life-threatening. Cardiorespiratory complications occurred in 18% of patients with complications, thromboembolic events in 5%, and surgical site infections in 8%. No cases of sepsis were recorded. Overall, 38% of patients with complications required an invasive procedure during hospitalization (Table 2). Bleeding was the most common complication, affecting 46% of patients with complications (corresponding to 4.2% of the total cohort). Additionally, 38% of patients who developed complications required an invasive procedure during hospitalization. Full descriptive data is available in Table 2.

## 4. Discussion

Metabolic and bariatric surgery remains the one of the most effective treatment for severe obesity, providing substantial benefits in sustained weight loss, remission of obesity associated medical problems, and reduction in cancer risk [7,8,9,10]. Although the overall safety profile of bariatric procedures has improved considerably over time, postoperative complications continue to pose clinically meaningful challenges. In our cohort, the overall complication rate was 9.1%, with 38% of affected patients requiring invasive intervention, highlighting the continued importance of accurate perioperative risk assessment and patient optimization.

The complication rate observed in our study is broadly consistent with contemporary bariatric surgery literature [13,22,23]. Over the past two decades, advances in minimally invasive surgical techniques, anesthesia protocols, and perioperative care pathways have significantly improved the safety profile of metabolic and bariatric procedures [13,16,24]. Enhanced recovery protocols, improved patient selection, and multidisciplinary perioperative management have further contributed to reducing postoperative morbidity. Despite these improvements, complications still occur in a clinically meaningful minority of patients, emphasizing the importance of identifying individuals at increased risk who may benefit from intensified monitoring or tailored perioperative strategies.

Our analysis identified several independent predictors of early postoperative complications, including OSA and recent bariatric surgery within the past five years, either revision or conversion. These findings provide clinically relevant insights that may assist in optimizing perioperative risk stratification and guiding individualized patient management.

OSA emerged as the strongest predictor of postoperative complications in our model, with an odds ratio of 1.93. This finding is consistent with prior literature, including the meta-analysis by Sun et al. [25], which demonstrated significantly increased risks of respiratory (OR = 1.91; 95% CI 1.54–2.36) and cardiac complications (OR = 1.74; 95% CI 1.25–2.42) in patients with OSA. The severity of OSA has also been associated with increased rates of intensive care unit admission, postoperative delirium, and prolonged hospital stay. These observations support the importance of routine preoperative screening and optimization of OSA management prior to bariatric surgery.

Several physiological mechanisms may explain the association between OSA and increased postoperative morbidity. Intermittent hypoxia, systemic inflammation, and increased cardiovascular stress may impair perioperative respiratory function and increase susceptibility to cardiopulmonary complications [26,27]. Early identification of OSA and appropriate perioperative management, including the use of continuous positive airway pressure (CPAP), may therefore represent important strategies for reducing postoperative risk in this patient population.

Patients who had undergone bariatric surgery within the preceding five years demonstrated double the odds of postoperative complications. Revisional bariatric procedures are inherently more complex than primary operations due to altered anatomy, adhesions, and scar tissue formation. These factors may increase operative complexity, prolong operative time, and raise the risk of intraoperative injury or postoperative complications. Previous studies, including those by Wysocki et al. [28] and Al-Mazrou et al. [29], have similarly shown elevated risks with revisional surgery (OR = 1.89; 95% CI: 1.06–3.34 and OR = 1.66; 95% CI: 1.43–1.94 respectively). Recent operations are likely associated with greater technical difficulty and perioperative challenges [28,29,30].

Pharmacologically treated type 2 diabetes demonstrated a non-significant trend toward an increased risk of postoperative complications. Chronic hyperglycemia has been linked to impaired immune function, reduced tissue perfusion, and delayed wound healing, all of which may contribute to postoperative morbidity [31]. Although not statistically significant in our cohort, the observed trend toward increased postoperative complications among patients with pharmacologically treated type 2 diabetes is in line with American Diabetes Association guidance identifying perioperative hyperglycemia as a modifiable risk factor for adverse surgical outcomes [31]. Optimization of glycemic control prior to surgery may therefore represent an important modifiable factor for improving perioperative outcomes in patients undergoing bariatric procedures.

We reviewed previously published studies (Table A1) and compared them to our results. our complication rate of 9.1% is slightly higher yet comparable to those reported by Iranmanesh et al. [32] (12.5%) and Zengin et al. [33] (32.8% in a smaller prospective cohort). In contrast, larger-scale database studies reported lower complication rates (Liang et al. [30] 3.79%, Chen et al. [34] 3.2%, Stenberg et al. [23] 3.4%). Our observed bleeding incidence (4.2% of the total cohort and 46% among complicated cases) significantly exceeds the bleeding rates reported by Sebastian et al. [35] (0.2–0.6%). These variations likely reflect differences in patient populations and surgical techniques.

Risk stratification represents a critical component of perioperative planning in bariatric surgery. Identifying patients at increased risk of complications allows clinicians to implement targeted strategies, including preoperative optimization of obesity associated conditions, enhanced intraoperative vigilance, and closer postoperative monitoring. In high-risk patients, multidisciplinary collaboration between surgeons, anesthesiologists, and medical specialists may further improve surgical outcomes.

The use of an unpenalized logistic regression model with standard maximum likelihood estimation allows valid statistical inference and ensures that the reported confidence intervals appropriately reflect predictor-specific uncertainty.

This study has several limitations. First, its retrospective design may introduce bias related to incomplete documentation or unmeasured confounding variables. Second, the analysis was conducted at a single tertiary referral center, which may limit the generalizability of the findings to other institutions or healthcare systems. Additionally, certain potentially relevant variables- such as socioeconomic factors, psychological comorbidities, and nutritional status- were not available in the dataset and therefore could not be included in the analysis. Nevertheless, the relatively large cohort and the consistency of our findings with previously published studies support the robustness of the results.

Taken together, our findings highlight several clinically relevant predictors of early postoperative complications following metabolic and bariatric surgery. Incorporating factors such as OSA, recent previous bariatric surgery, and diabetes into preoperative risk assessment models may facilitate more personalized perioperative management and ultimately contribute to improving patient outcomes and maintaining the favorable safety profile of bariatric surgery.

## 5. Conclusions

This study identified OSA and recent bariatric surgery within the past five years as key predictors of early postoperative complications following metabolic and bariatric surgery. These findings highlight the importance of comprehensive preoperative evaluation and careful patient selection when planning bariatric procedures. Early identification of high-risk patients may enable targeted perioperative optimization, including improved management of associated medical problems, enhanced monitoring, and individualized surgical planning. Incorporating these predictors into clinical risk stratification models and preoperative assessment protocols may help guide decision-making, improve patient counseling, and ultimately enhance surgical safety and outcomes. In addition, these findings may support the development of more personalized perioperative management strategies for patients undergoing bariatric surgery. Future research should aim to validate these predictors in larger multicenter cohorts and investigate targeted interventions that may further reduce postoperative complications in high-risk populations.

## Figures and Tables

**Table 1:** Baseline Characteristics and Univariate Comparison Between Patients With and Without Postoperative Complications.

Feature	Total (n = 927)	No Complication (n = 843)	Complication (n = 84)	*p*-Value
Gender	96 (10%)	90 (11%)	6 (7%)	0.409
Age at time of surgery	41.7 +/- 13.2	41.4 +/- 13.3	45.3 +/- 12.2	0.01
BMI on the day of hospitalization	41.5 +/- 9.3	41.6 +/- 9.4	39.7 +/- 7.6	0.063
Current Smoker	224 (24%)	200 (24%)	24 (29%)	0.392
Cigarettes per day	14.0 +/- 5.2	14.0 +/- 5.0	14.2 +/- 6.5	0.688
Years of smoking	17.4 +/- 6.4	17.3 +/- 5.9	18.3 +/- 10.1	0.181
Hypertension	302 (33%)	269 (32%)	33 (39%)	0.21
DM	241 (26%)	213 (25%)	28 (33%)	0.14
DM treated with medication	165 (18%)	143 (17%)	22 (26%)	0.05
Dyslipidemia	286 (31%)	259 (31%)	27 (32%)	0.885
Stress urinary incontinence	196 (21%)	178 (21%)	18 (21%)	0.942
OSA treated with medication	61 (7%)	54 (6%)	7 (8%)	0.654
Dyslipidemia treated with medication	147 (16%)	132 (16%)	15 (18%)	0.712
Asthma	77 (8%)	67 (8%)	10 (12%)	0.296
Asthma treated with medication	61 (7%)	54 (6%)	7 (8%)	0.654
Hypertension treated with medication	260 (28%)	231 (27%)	29 (35%)	0.208
GERD	200 (22%)	177 (21%)	23 (27%)	0.223
GERD treated with medication	124 (13%)	110 (13%)	14 (17%)	0.447
Arthralgia	426 (46%)	388 (46%)	38 (45%)	0.981
OSA	174 (19%)	150 (18%)	24 (29%)	0.023
How many floors can climb in a row	6.5 +/- 18.4	6.5 +/- 18.4	6.3 +/- 18.0	0.923
Previous abdominal surgery	335 (36%)	301 (36%)	34 (40%)	0.454
Previous bariatric surgery	238 (26%)	208 (25%)	30 (36%)	0.038
Less than 5 years from previous bariatric surgery	71 (8%)	57 (7%)	14 (17%)	0.002
Addition to surgery	173 (19%)	159 (19%)	14 (17%)	0.73
Type of surgery: One Anastomosis Gastric Bypass (Omega loop, Mini)	500 (54%)	463 (55%)	37 (44%)	0.073
Type of surgery: Sleeve gastrectomy	282 (30%)	256 (30%)	26 (31%)	0.989
Type of surgery: Roux-en-Y gastric bypass	139 (15%)	118 (14%)	21 (25%)	0.011
Type of surgery: One Anastomosis Duodenal switch (Omega loop)	5 (1%)	5 (1%)	0 (0%)	0.942
Surgery Length	106.0 +/- 51.5	104.9 +/- 48.8	117.4 +/- 72.9	0.034
Length of stay	3.9 +/- 2.9	3.4 +/- 1.4	9.4 +/- 6.4	0

**Table 2:** Distribution and Severity of Postoperative Complications According to the Clavien-Dindo Classification.

Feature	Complication (n = 84)
Clavien-Dindo Grade 1	51 (60%)
Clavien-Dindo Grade 2	9 (11%)
Clavien-Dindo Grade 3	15 (18%)
Clavien-Dindo Grade 4	9 (11%)
Leak/intra-abdominal abscess	20 (24%)
Life-threatening Leak/intra-abdominal abscess	5 (6%)
Surgical site infection	6 (8%)
Life-threatening surgical site infection	0 (0%)
Thromboembolic event	4 (5%)
Life-threatening thromboembolic event	1 (1%)
Cardio/Respiratory complication	15 (18%)
Life-threatening cardio/respiratory complication	3 (3%)
Sepsis	0 (0%)
Life-threatening Sepsis	0 (0%)
Bleeding	39 (46%)
Invasive procedure during hospitalization	32 (38%)
Mortality (Perioperative and 90-day)	0 (0%)

## Data Availability

The data sets used and/or analyzed during the current study are available from the corresponding author upon reasonable request.

## Abbreviations

The following abbreviations are used in this manuscript:

OSA	Obstructive sleep apnea
OR	Odds Ratio

## Appendix A

**Table A1:** Summary of Published Studies Reporting Risk Factors for Complications Following Bariatric Surgery.

Article Title, Author Name, Year of Publication	Number of Participants	Type of Study	Type of Bariatric Surgeries	Complication Rate	Overall Mortality Rate	Risk Factors Identified
Liang et al. [30] 2025	323,066	Database analysis	SG, RYGB	3.79%	Not specified	Advanced age Anemia Cardiovascular disease Coagulation disorders RA Drug abuse Electrolyte imbalance Neurological condition RF PUD
Hon et al. [20] 2025	Not specified	Systematic review	Various primary bariatric surgeries	Not specified	Reported for older age groups: 2.62 × risk	Older age Male sex Black/African American race Low financial status Marital status
Sebastian et al. 2024 [35]	1,002,505	Database analysis (MBSAQIP registry)	SG, RYGB	0.2–0.6% bleeding	Not specified	Anticoagulation Renal insufficiency DVT/PE history BMI ≤ 40
Iranmanesh et al. [32] 2023	1276	Multicenter retrospective cohort study	RYGB	12.5%	0.1%	Male sex ASA score > 2
Zengin et al. [33] 2022	67	Prospective cohort study	SG, GB	32.8%	Not reported	High baseline intra-abdominal pressure
Nijland et al. [36] 2020	1669	Retrospective cohort study	RYGB, SG	8.3% complications	0%	Depression Sleeve gastrectomy Postoperative complications
Chang et al. [22] 2018	107,874	Systematic review and meta-analysis	GB, LAGB, SG	Anastemitic Leak 1.15%, MI 0.37%, PE 1.17%,	0.12–0.37%	Age Hypertension Smoking history Male sex
Lynn et al. [37] 2018	485	Prospective chorot study	RYGB, SG	4.7%	0% (inpatient)	None significantly different between groups
Chen et al. [34] 2015	113,898	Database analysis (NSQIP registry)	GB, LAGB, SG	3.2%	0.11% (30 days)	BMI ≥50 steroid use surgery type predischarge complications
Stenberg et al. [23] 2014	26,173	Database analysis (Scandinavian obesity surgery registry)	RYGB	3.4%	0.04% (90 days)	Intraoperative adverse events Conversion to open surgery Low hospital volume
